# Supplementary material for: Rab5-mediated endosome formation is regulated at the trans-Golgi network
Source: Commun Biol. 2019 Nov 15;2:419. doi: 10.1038/s42003-019-0670-5 (PMC6858330; doi:10.1038/s42003-019-0670-5)
Supplement: Supplementary file 1 — Supplementary Infomation [file 42003_2019_670_MOESM1_ESM.pdf]

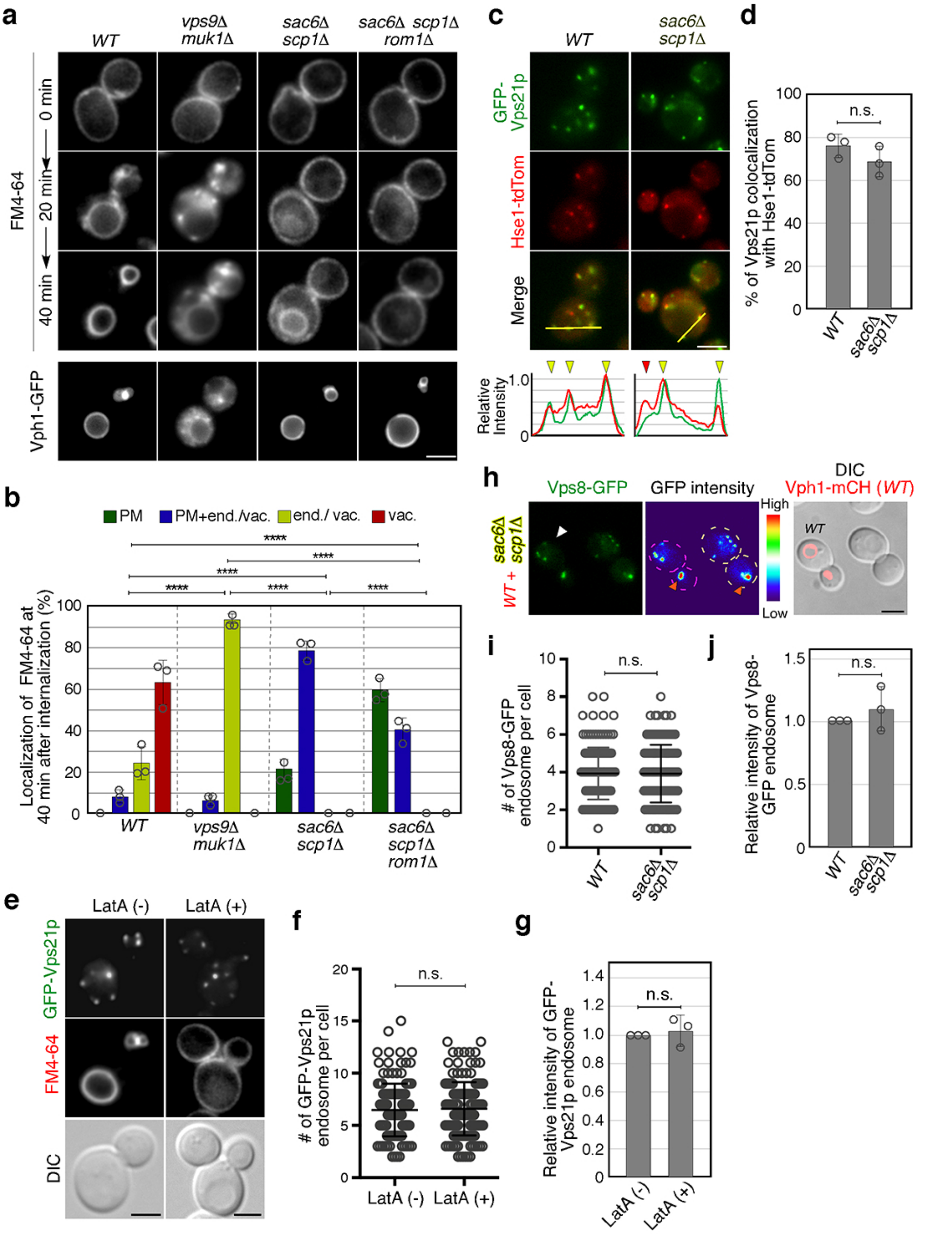

Supplementary Figure 1. Defective endocytosis does not affect the localization of Vps21p and Vps8p. (a) Effect of the deletion of Rab5-specific GEFs or endocytosis-related proteins on the internalization of FM4-64 or Vhp1-GFP transport to the vacuole. The images were acquired at 0, 20 and 40 min after washing out unbound FM4-64 (upper panels). (**b**) Quantification of the localization of FM4-64 in the indicated cells at 40 min after internalization. The compartments were categorized into four classes; plasma membrane only (PM), PM and endosome and/or vacuole (PM+end./vac.), endosome and/or vacuole (end./vac.) and vacuole only (vac.). (**c**) Localization of GFP-Vps21p and tdTomato-tagged Hse1p in wild-type (*WT*) and *sac6*Δ *scp1*Δ cells. Representative intensity profiles of GFP-Vps21p and Hse1-tdTomato along the yellow line in the merged images are indicated in the lower graphs. Yellow arrowheads indicate colocalization. (**d**) The percentages of colocalization were calculated as the ratio of GFP-Vps21p (n > 100) colocalizing with Hse1-tdTomato positive puncta in each experiment. Data show mean ± SEM from three independent experiments. Unpaired *t*-test with Welch's correction. (**e**) The effect of Latrunculin A (LatA) on the localization of Vps21p. Cells expressing GFP-Vps21p were incubated with 400 μM LatA and 200 μM FM4-64 for 40 min at 25°C and observed after washing out unbound FM4-64. (**f** and **g**) Quantification of the (f) number or (g) fluorescence intensity of GFP-Vps21p positive endosomes displayed in (e). (**h**) Localization of Vps8-GFP in *WT* and mutant cells. Cells expressing Vps8-GFP were grown to early- to mid-logarithmic phase in SD+SC medium at 25°C, mixed and acquired in the same images. Fluorescence images or heat maps showing GFP levels are shown in the upper (Vps8-GFP) or middle row (GFP intensity), respectively. *WT* or mutant cells are indicated with red or yellow dashed lines, respectively. *WT* cells are labeled by the expression of Vph1-mCherry (red) which is shown in the lower images overlaid with DIC images. (**i** and **j**) Quantification of the number (i) or fluorescence intensity (j) of Vps8-GFP positive endosomes displayed in (h). Data show mean ± SD with 150 cells (**f** and **i**) or mean ± SEM from three independent experiments with 100 endosomes (g and j). Unpaired *t*-test with Welch's correction (d, f, g, i and j). n.s., not statistically significant. ****p <0.0001, chi-square test for trend (b). Scale bars, 2.5 μm.

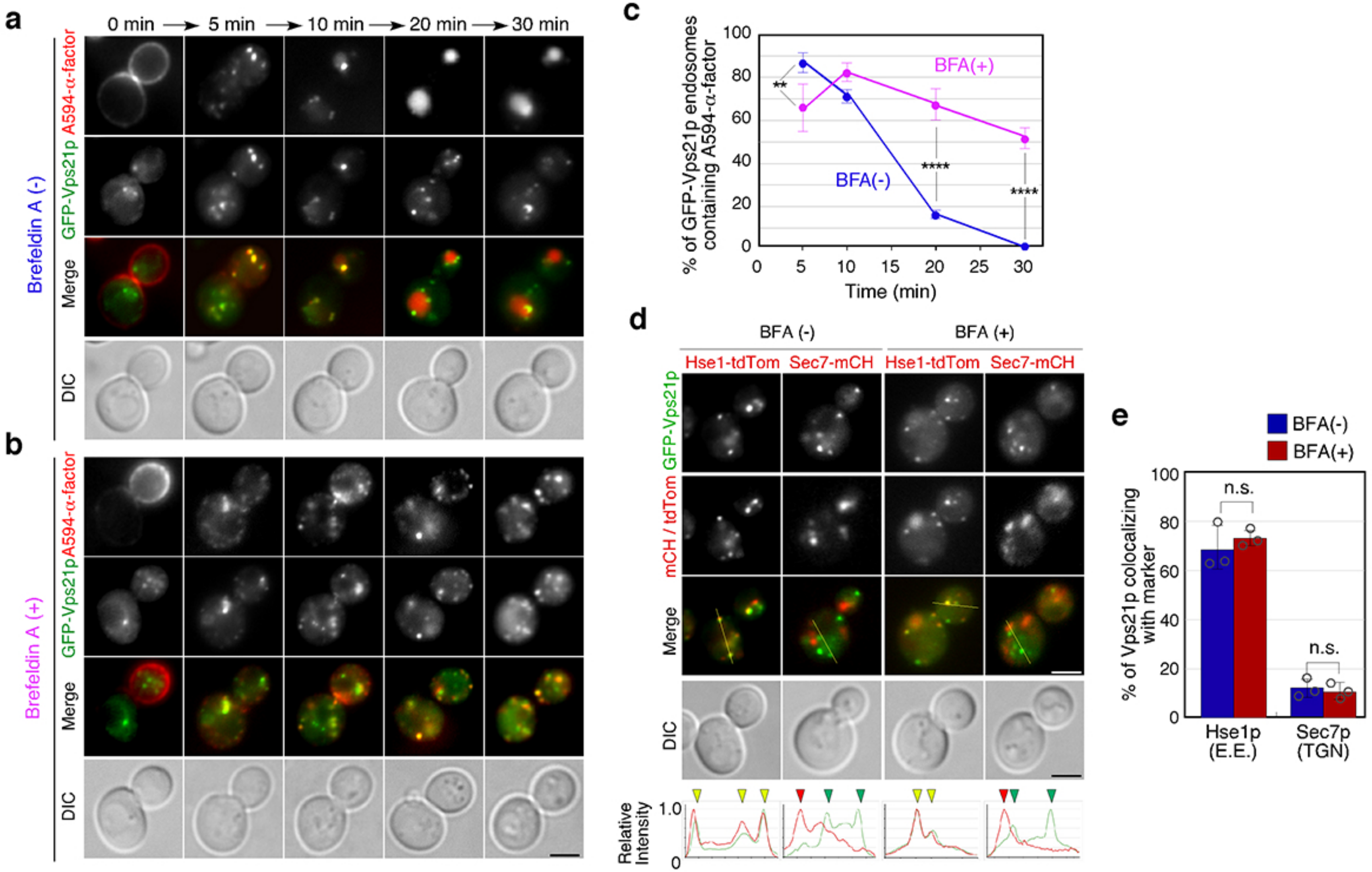

Supplementary Figure 2. Effect of Brefeldin A on Vps21-mediated vesicle formation and trafficking. (**a** and **b**) The spatio-temporal localization of Alexa-α-factor and GFP-Vps21p in Brefeldin A (BFA)-treated cells. Cells expressing GFP-Vps21p were labeled with Alexa-α-factor in the absence (a) or presence (b) of 100 μg m^{-1} L^{-1} BFA. The images were acquired at 0, 5, 10, 20 and 30 min after washing out unbound Alexa-α-factor and incubating the cells at 25°C. (**c**) Quantification of the colocalization of GFP-Vps21p with Alexa-α-factor at each time point, calculated as the ratio of Alexa-α-factor localized in GFP-Vps21p positive compartments (n > 100) in each experiment. Error bars represent the SD from three independent experiments. (**d**) Colocalization of GFP-Vps21p and Hse1p-tdTomato (Hse1-tdTom) or Sec7p-mCherry (Sec7-mCH) in in the absence (BFA (-)) or presence (BFA (+)) of 100 μg m^{-1} L^{-1} BFA. Representative intensity profiles of GFP-Vps21p and Hse1-tdTom or Sec7-mCH along the yellow line in the merged images are indicated in the lower graphs. Yellow or red/green arrowheads indicate the presence or absence of colocalization, respectively. (e) The percentages of colocalization were calculated as the ratio of mCH/tdTom-tagged marker (n = 100) colocalizing with GFP-Vps21p positive puncta in each experiment. $**p < 0.01$, $****p < 0.0001$, two-way ANOVA with Tukey's post-hoc test (e). Scale bar in all panels, 2.5 μm.

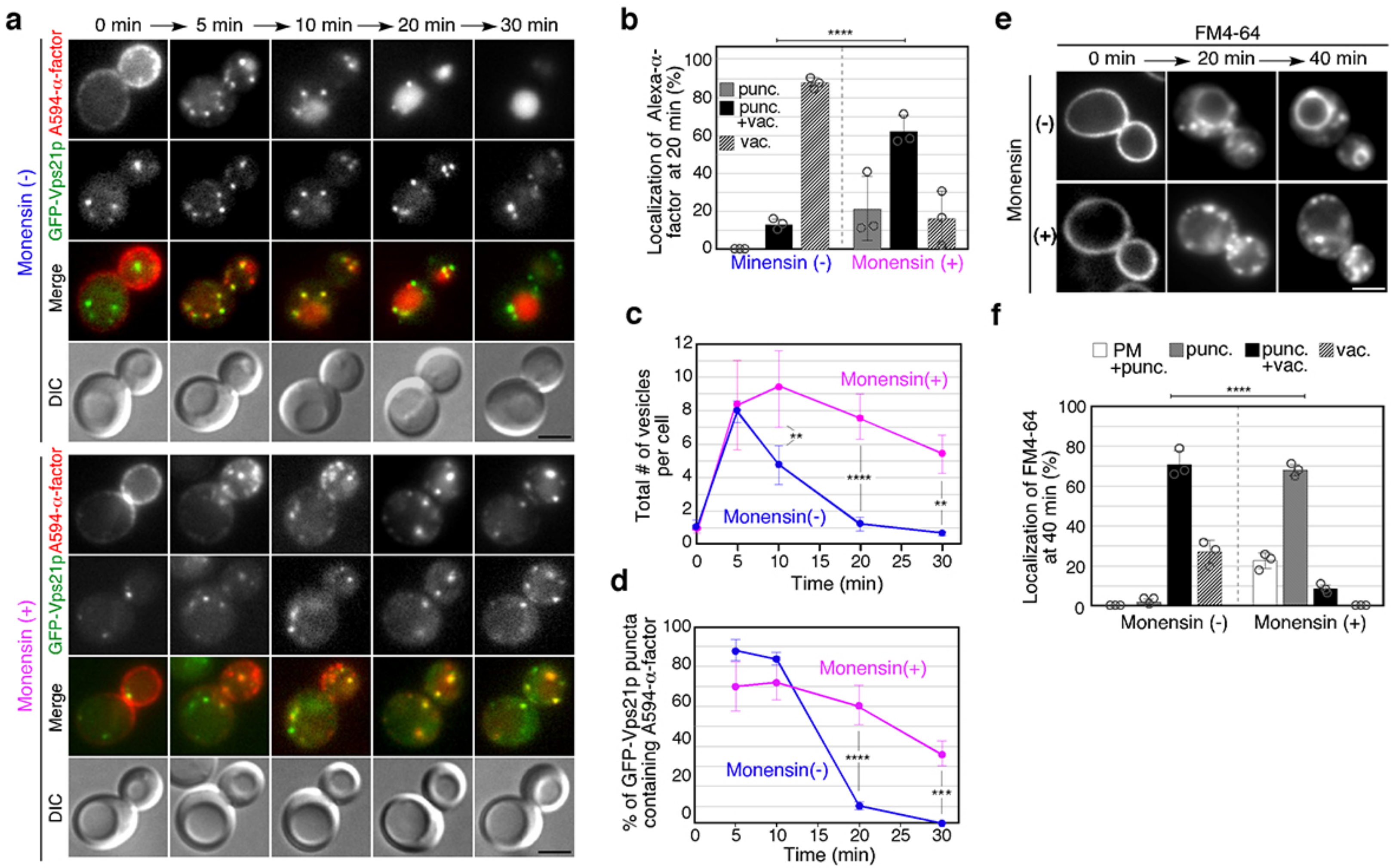

Supplementary Figure 3. Effect of Monensin on Vps21-mediated vesicle formation and trafficking. (**a**) The spatio-temporal localization of Alexa-α-factor and GFP-Vps21p in Monensin-treated cells. Cells expressing GFP-Vps21p were labeled with Alexa-α-factor in the absence or presence of 50 μM Monensin. The images were acquired at 0, 5, 10, 20 and 30 min after washing out unbound Alexa-α-factor and incubating the cells at 25ºC. (**b**) Quantification of the localization of Alexa-α-factor in the indicated cells at 20 min after internalization. The localization of Alexa-α-factor was categorized into three classes; puncta only (punc.), puncta and vacuole (punc. vesi.+vac.), and vacuole only (vac.). Data show mean ± SEM from three independent experiments, with >100 cells counted for each strain per experiment. (**c**) Quantification of the number of Alexa-α-factor positive puncta displayed in (a). (**d**) Quantification of the colocalization of GFP-Vps21p with Alexa-α-factor at each time point, calculated as the ratio of Alexa-α-factor localized in GFP-Vps21p positive compartments (n>100) in each experiment. Error bars represent the SD from three independent experiments. (**e**) Effect of Monensin treatment on FM4-64 transport from the PM to the vacuole. After treatment of the cells with 50 μM Monensin, cells were labeled with 200 μM FM4-64 for 15 min on ice and observed at 0, 20, and 40 min after washing out unbound FM4-64 and incubating the cells at 25ºC. (**f**) Quantification of the localization of FM4-64 in the indicated cells at 40 min after internalization. PM and puncta (PM+ punc.), puncta only (punc.), puncta and vacuole (punc.+vac.), and vacuole only (vac.). Data show mean ± SEM with 100 puncta (b, c, and f) or 100 cytosol (a) from three independent experiments. ****p <0.0001, chi-square test for trend (b and f). *p <0.05, **p <0.01, ****p <0.0001, two-way ANOVA with Bonferroni's post-hoc test (c). *p <0.05, **p <0.01, ***p <0.001, ****p <0.0001, two-way ANOVA with Tukey's post-hoc test (d). Scale bar in all panels, 2.5 μm.

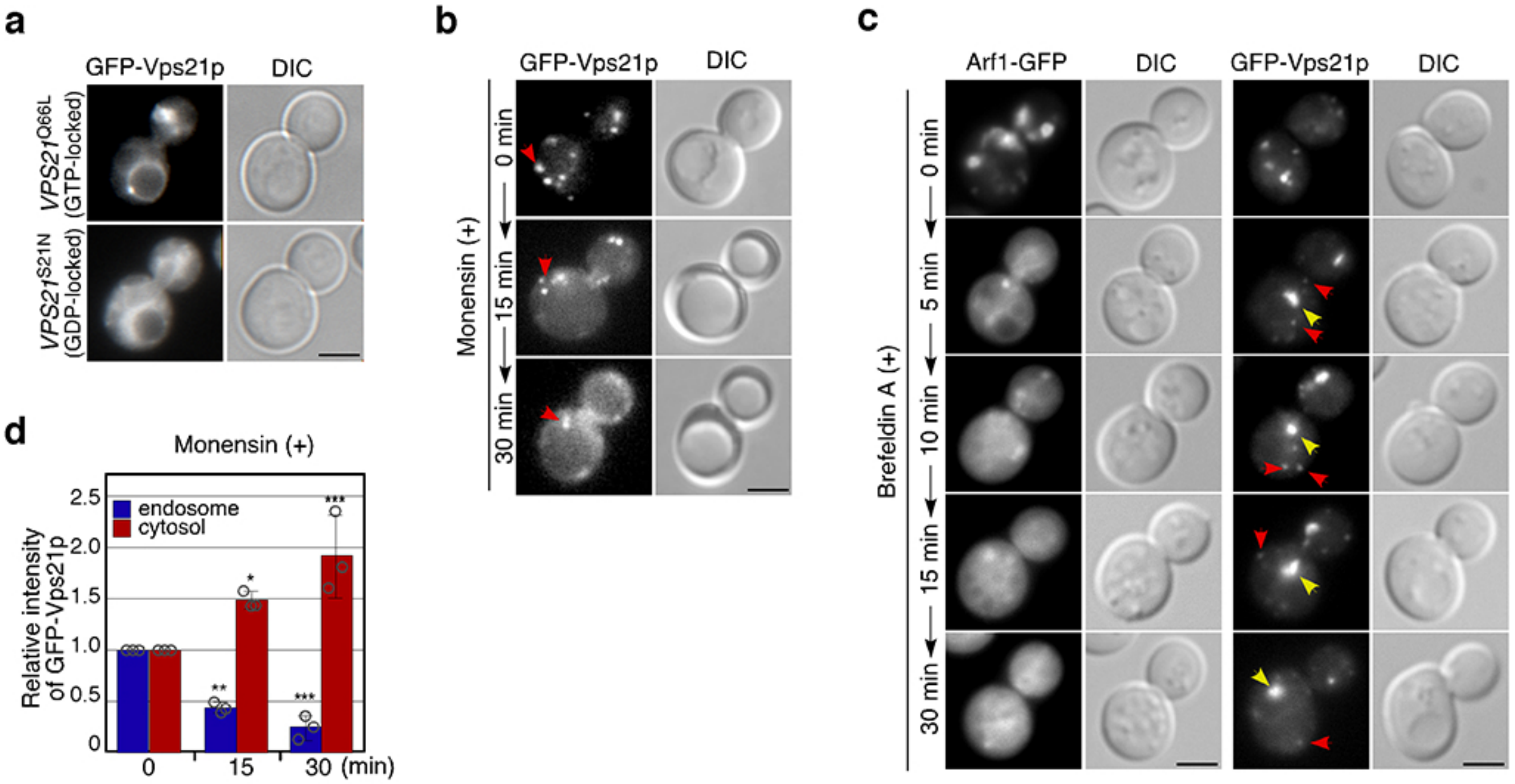

Supplementary Figure 4. Effect of inhibiting post-Golgi traffic on Vps21p localization. (**a**) Localization of the GFP-tagged constitutively active Vps21Q66L or constitutively inactive Vps21S21N mutant in cells. (b) Effect of Monensin on the localization of Vps21p. Cells expressing GFP-Vps21p were incubated with 50 μM Monensin at 25°C and observed at 0, 15 and 30 min after incubation. (**c**) Effect of BFA on the localization of Vps21p. Cells expressing Arf1-GFP or GFP-Vps21p were incubated with 100 μg m^{-1} L^{-1} BFA at 25°C and observed at 0, 5, 10, 15 and 30 min after incubation. Red arrows indicate the example of GFP-Vps21p endosomes and yellow arrows show aberrant accumulation of GFP-Vps21p. (**d**) Quantification of the fluorescence intensity of GFP-Vps21p at endosomes and in the cytosol. Data show the mean ± SEM of three experiments, with 100 cells counted at each time point per experiment. Data show mean ± SEM with 100 puncta (g). *p <0.05, **p <0.01, ***p <0.001, two-way ANOVA with Tukey's post-hoc test (g). Scale bar in all panels, 2.5 μm.

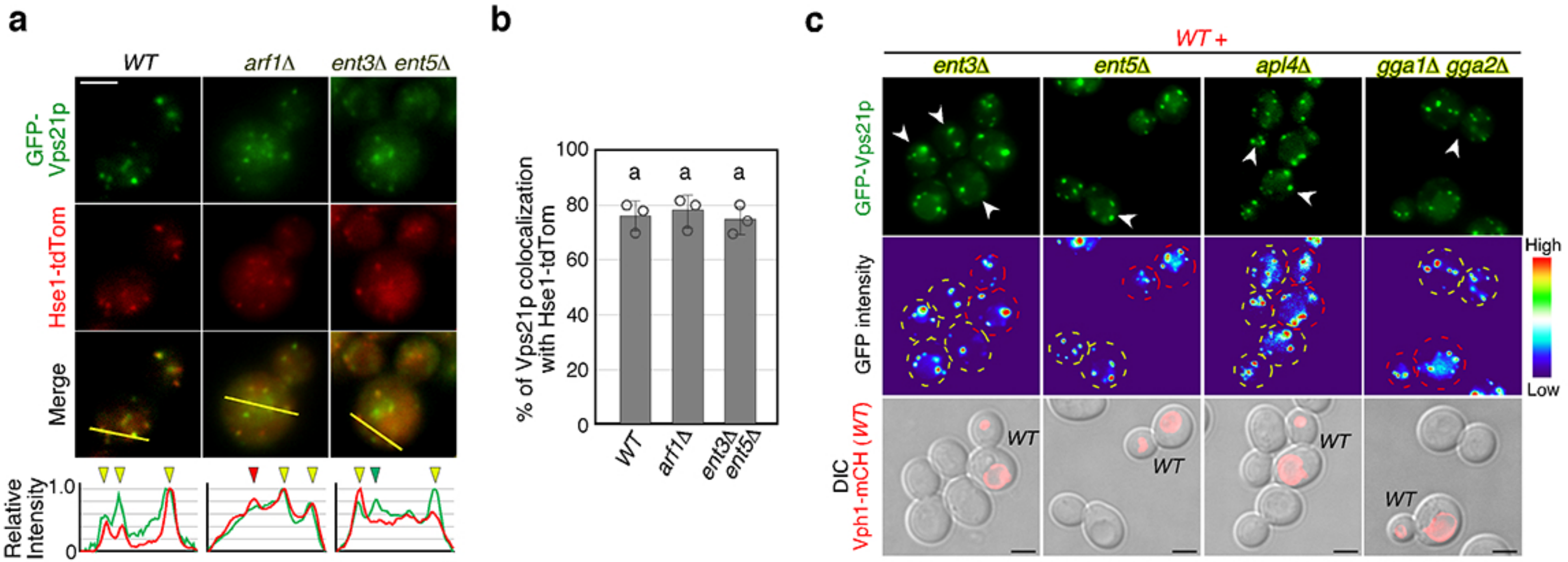

Supplementary Figure 5. Localization of Vps21p in cells lacking TGN-resident adaptor protein(s). (**a**) Localization of GFP-Vps21p and tdTomato-tagged Hse1p in wild-type (*WT*) and mutant cells. Representative intensity profiles of GFP-Vps21p and Hse1-tdTomato along the yellow line in the merged images are indicated in the lower graphs. Yellow or red/green arrowheads indicate the presence or absence of colocalization, respectively. (**b**) The percentages of colocalization were calculated as the ratio of Hse1-tdTomato colocalizing with GFP-Vps21p positive puncta (n = 100) in each experiment. Data show mean ± SEM from three independent experiments. Different letters indicate significant difference at $p < 0.05$, one-way ANOVA with Tukey's post-hoc test. (**c**) Localization of GFP-Vps21p (A) in wild-type (*WT*) and mutant cells. Fluorescence images (GFP-Vps21p), heat maps showing GFP levels (GFP intensity) are shown. *WT* cells are labeled by the expression of Vph1-mCherry (red) which is shown in the images overlaid with DIC images. Scale bars, 2.5 μm.

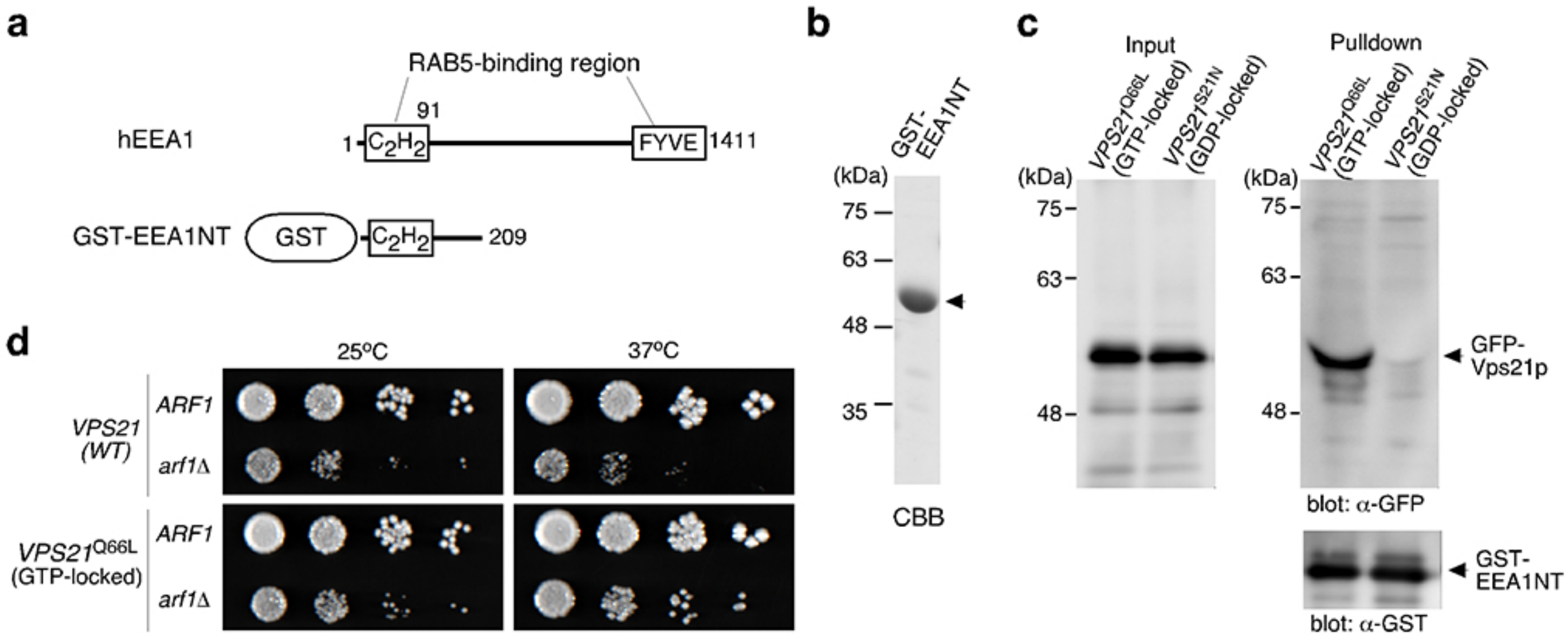

Supplementary Figure 6. Detection of the GTP-bound active form of Vps21p *in vitro* and complementation of the growth phenotype of *arf1*Δ cells by Vps21Q66L. (**a**) Schematic illustration of human Early Endosome Antigen 1 (EEA1) and its Rab5-binding regions. An N-terminal fragment (NT) of EEA1 was utilized as the bait for the pulldown of active Vps21p. (**b**) Purification of GST-EEA1NT. (**c**) Binding specificity of GST-EEA1NT to active Vps21p. Cells expressing GFP-tagged constitutively active Vps21Q66L or constitutively inactive Vps21S21N were lysed, and 6 μg of total cell lysate (4% input) was loaded and immunoblotted with an anti-GFP antibody (Input panel). Active Vps21p from 150 μg of total cell lysate was pulled down with the GST-tagged N terminal portion of human EEA1 (GST-EEA1NT) and probed with an anti-GFP antibody (Pulldown, α-GFP panel) or an anti-GST antibody (Pulldown, α-GST panel). (**d**) Complementation of the growth phenotype of *arf1*Δ cells by constitutively active Vps21Q66L. A dilution series of cells expressing GFP-tagged wild-type Vps21p or constitutively active Vps21Q66L was spotted on YPD plates and incubated at the indicated temperature for 2 or 3 days.

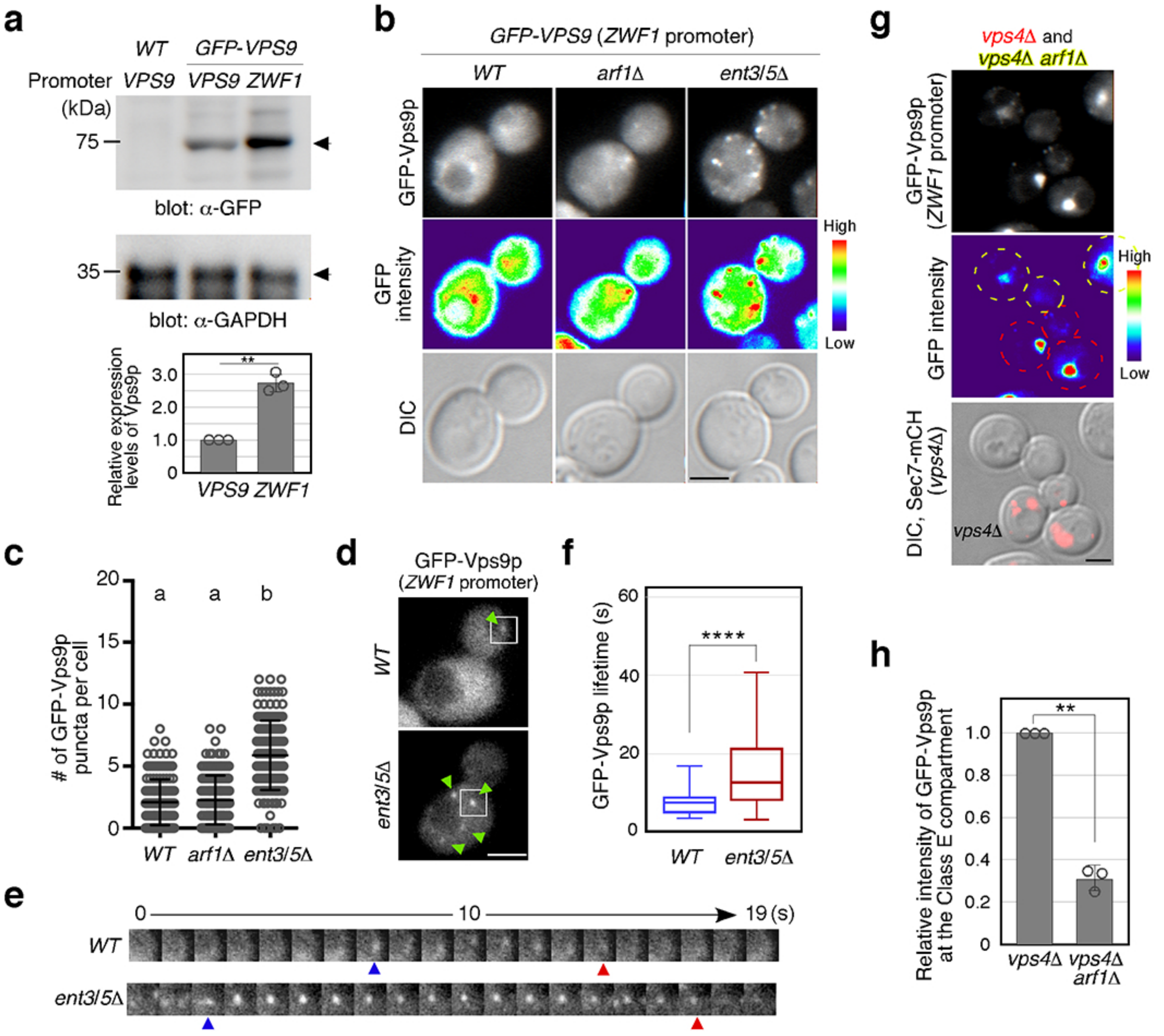

Supplementary Figure 7. Arf1p and Ent3p/5p-dependent localization of Vps9p at the endosomes. (**a**) Immunoblots showing the expression levels of GFP-tagged Vps9p under the control of its endogenous promoter in *WT* or the indicated mutant cells. Vps9p was tagged with GFP at the N terminus and expressed under the control of the authentic promoter from the endogenous locus. Total cell lysate were loaded and immunoblotted with an anti-GFP antibody (α-GFP panel). GAPDH was used as loading control (α-GAPDH panel). Graph showed mean ± SEM from three independent experiments. (**b**) Localization of GFP-Vps9p in *WT* and the indicated mutant cells. Vps9p was tagged with GFP at the N terminus and expressed under the control of *ZWF1* promoter from the endogenous locus. Fluorescence images (GFP-Vps9p panels), heat maps showing GFP levels (GFP intensity panels) and DIC images (DIC panels) were shown. (**c**) Quantification of the number of GFP-Vps9p puncta displayed in (b). (**d** and **e**) Dynamic behavior of GFP-Vps9p under the control of the *ZWF1* promoter in indicated strains. The time series of the regions in the boxed area in (d) were shown in (e). Blue and red arrowheads indicate appearing and disappearing points of GFP-Vps9p (e). (**f**) Graph showed average lifetime of GFP-Vps9p in the indicated strains. n = 100 puncta for each strain. Top and bottom bars are the 95% confidence limits. (**g**) Localization of GFP-Vps9p in *vps4*Δ and *vps4*Δ *arf1*Δ cells. (**h**) Quantification of the fluorescence intensity of GFP-Vps9p-accumulated compartment displayed in (g). Data show mean ± SD with 150 cells (f) or mean ± SEM with 100 puncta (h) from three independent experiments. $^{**}p < 0.01$, $^{****}p < 0.0001$, unpaired *t*-test with Welch's correction (a, f, and h). Different letters indicate significant difference at $p < 0.05$, one-way ANOVA with Tukey's post-hoc test (c). Scale bar in all panels, 2.5 μm.

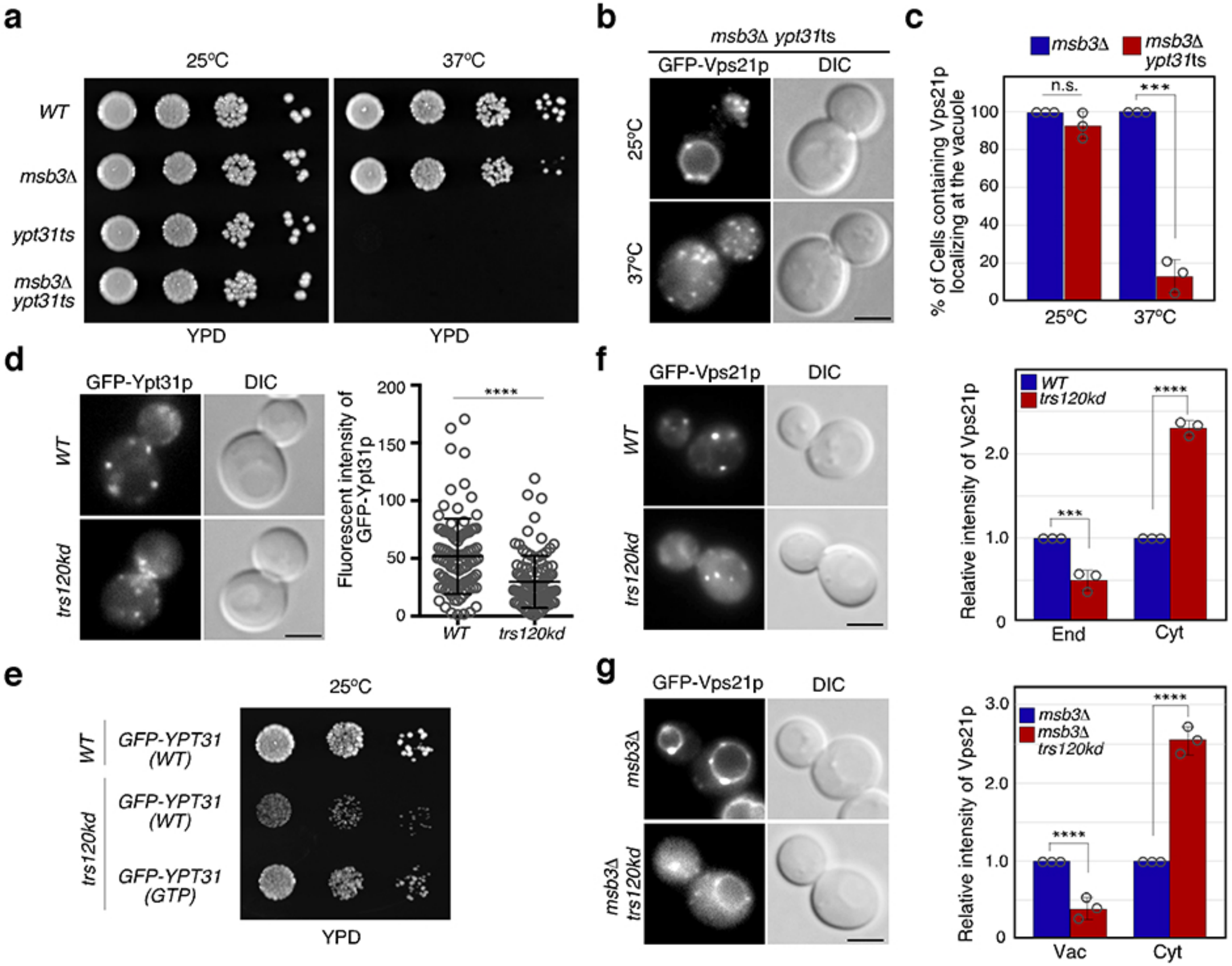

Supplementary Figure 8. Ypt31/32p-dependent localization of Vps21p at the endosomes. (**a**) Plates showing the growth phenotype of indicated strains. Cells were plated on YPD plates and incubated at 25ºC or 37ºC, respectively. (**b**) Localization of GFP-Vps21p in *msb3*Δ *ypt31ts* cells. After being grown to early to mid-logarithmic phase, cells were incubated for 90 m either at 25ºC or 37ºC and then observed by fluorescence microscopy and DIC. (**c**) Quantification of the percentages of cells containing Vps21p localizing at the vacuole at 25ºC or 37ºC (n = 100 for each). (**d**) Localization of GFP-Ypt31p in *WT* and *trs120kd* cells. Graph shows quantification of the fluorescence intensity of GFP-Ypt31p at punctate structures (n = 100 for each). (**e**) Plates showing the growth phenotype of indicated strains. wild-type; *WT*, Trs120p knockdown; *trs120kd*. Cells were plated on YPD plates and incubated at 25ºC. (**f**) Localization of GFP-Vps21p in *WT* and *trs120kd* cells. Graph shows quantification of the fluorescence intensity of GFP-Vps21p at the endosome (End) or the cytoplasm (Cyt) (n = 100 for each). (**g**) Localization of GFP-Vps21p in *msb3*Δ and *trs120kd msb3*Δ cells and quantification of the fluorescence intensity of GFP-Vps21p at the vacuolar membrane (Vac) or the cytoplasm (Cyt) (n = 100 for each). Data show mean ± SEM with 100 cells (c) or 100 puncta (d) from three independent experiments. ***p < 0.001, n.s., not significant, one-way ANOVA with Tukey's post-hoc test (c). ****p <0.0001, unpaired t-test with Welch's correction (d). ***p <0.001, ****p <0.0001, two-way ANOVA with Bonferroni's post-hoc test (f and g).

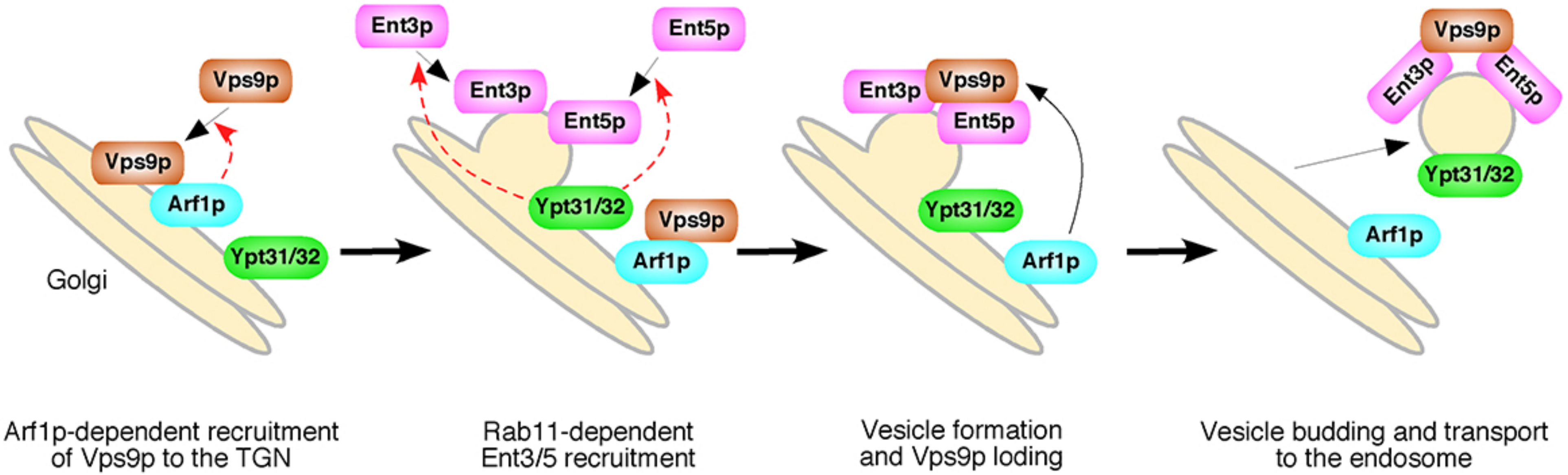

Supplementary Figure 9. Model for the regulation of Vps9p transport from the TGN to the endosome. Vps9p is recruited to the TGN dependently on Arf1p. Ytp31p/32p regulate the recruitment of Ent3p/5p adaptors to the TGN, and promote post-Golgi vesicle formation. Then, Vps9p is loaded on the vesicle containing Ent3p/5p adaptors, and the vesicle buds off from the TGN and is transported to the endosome. The details are described in the text.

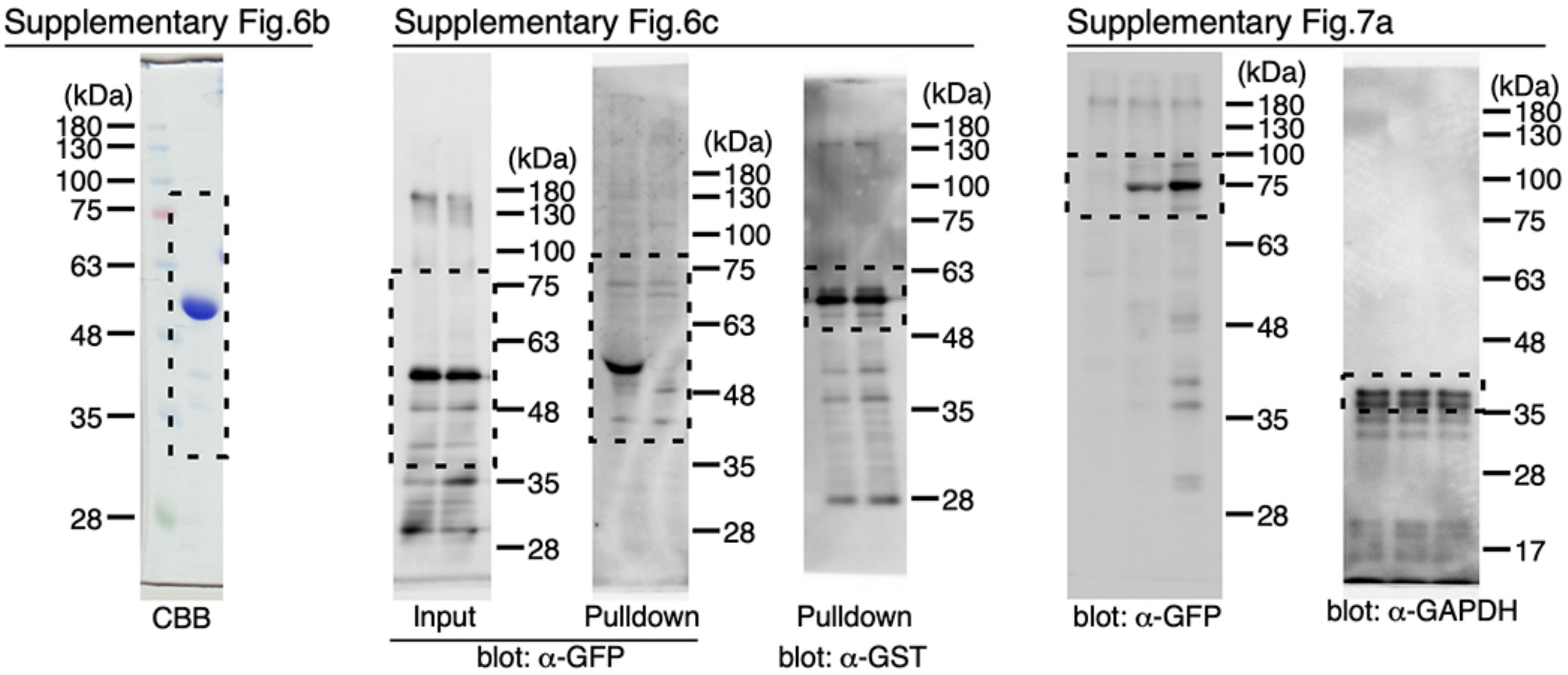

Supplementary Figure 10. Full uncropped gel/blot images in the supplementary figures.

Fig.3e

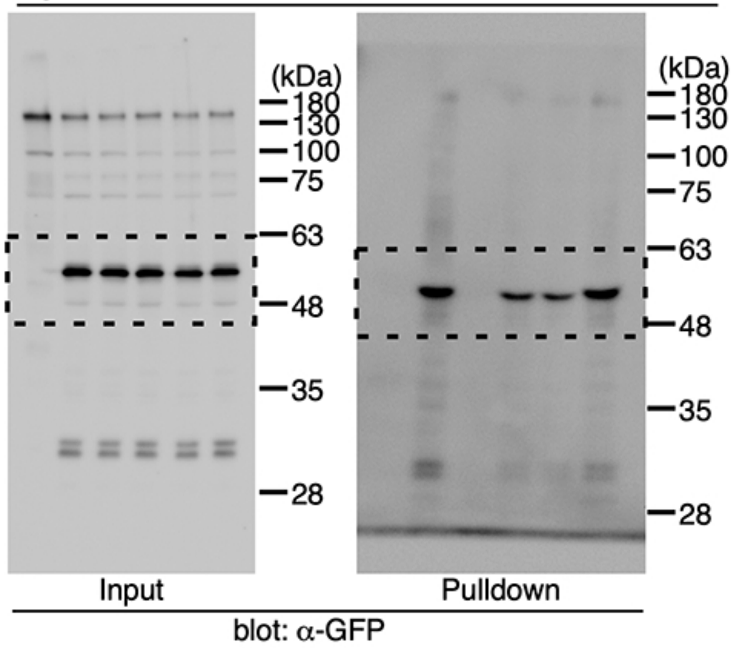

Fig.4a

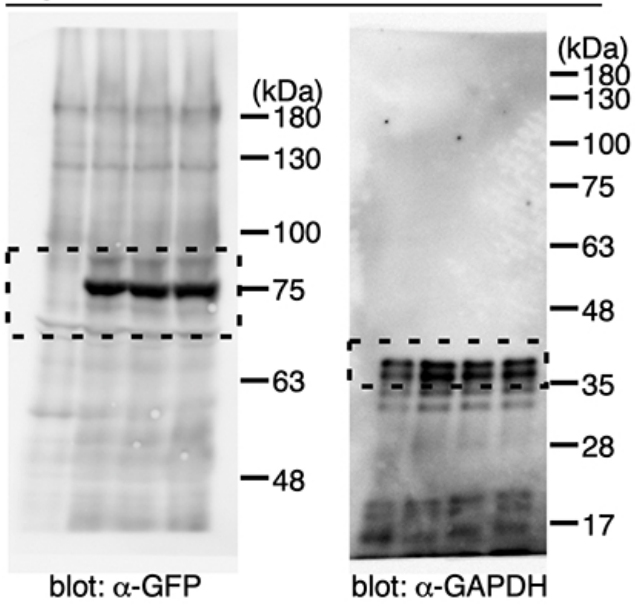

Fig.4i

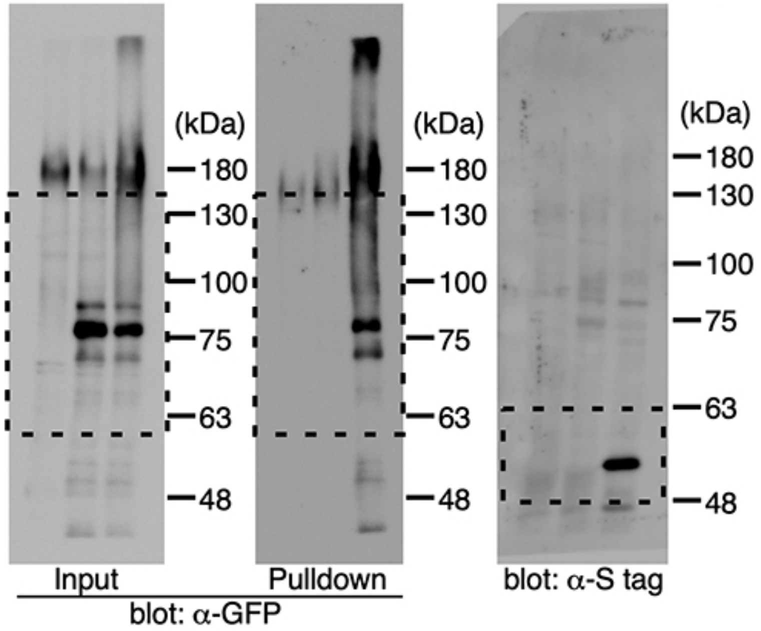

Fig.6f

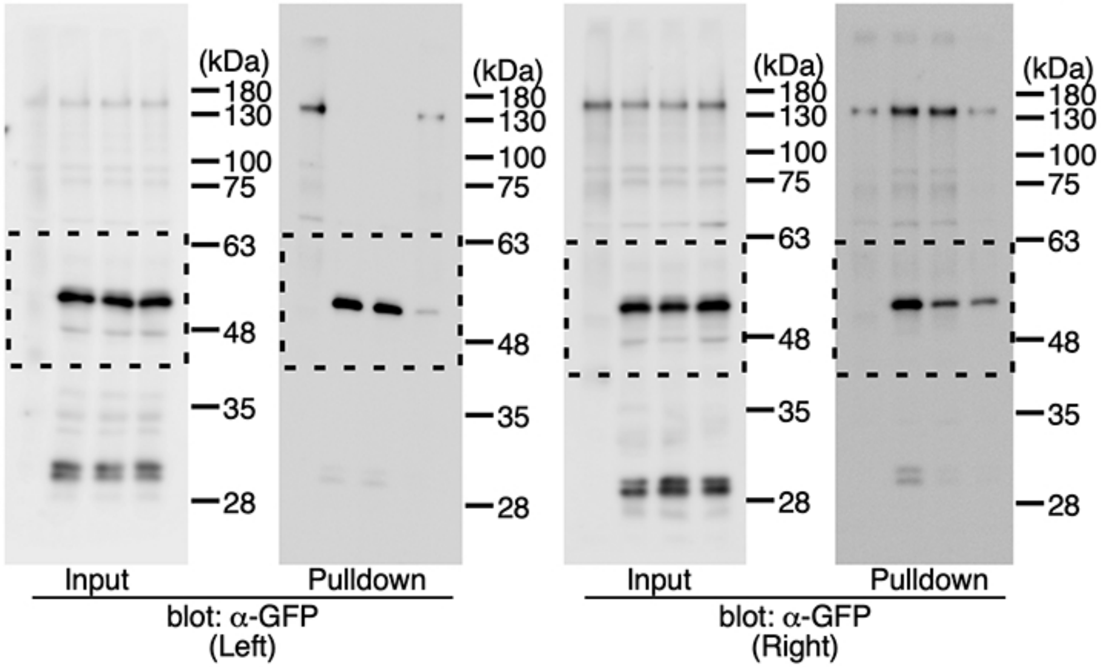

Supplementary Figure 11. Full uncropped blot images in the main figures.

Supplementary Table1. Yeast Strains used in this study

Strain	Genotype	Source
JJTY0568	*Mat***a** *his3-Δ200 leu2-3, 112 ura3-52 bar1Δ::LEU2 sla2Δ::URA3*	Toshima Lab
JJTY1178	*Mat***a** *his3Δ1 leu2Δ0 ura3Δ0 lys2Δ0 sac6Δ::KanMX6 scp1Δ::LEU2 bar1Δ::URA3*	Toshima Lab
JJTY1219	*Mat***a** *his3Δ1 leu2Δ0 ura3Δ0 lys2Δ0*	Toshima Lab
JJTY2006	*Mat***a** *his3Δ1 leu2Δ0 ura3Δ0 lys2Δ0 GFP-VPS21::HIS3 bar1Δ::LEU2*	Toshima Lab
JJTY1475	*Mat***a** *his3Δ1 leu2Δ0 met15Δ0 ura3Δ0 GFP-VPS21::HIS3*	this study
JJTY3485	*Mat***a** *his3Δ1 leu2Δ0 ura3Δ0 met15Δ0 vps9Δ::KanMX6 GFP-VPS21::HIS*	this study
JJTY3636	*Mat***a** *his3Δ1 leu2Δ0 ura3Δ0 lys2Δ0 GFP-YPT31::HIS3*	this study
JJTY3638	*Mat***a** *his3Δ1 leu2Δ0 ura3Δ0 lys2Δ0 GFP-VPS21::HIS3*	this study
JJTY3665	*Mat***a** *his3Δ1 leu2Δ0 ura3Δ0 lys2Δ0 ypt32Δ::KanMX6 YPT31(K127N)::URA3 GFP-VPS21::HIS3*	this study
JJTY3667	*Mat***a** *his3Δ1 leu2Δ0 ura3Δ0 lys2Δ0 ypt32Δ::KanMX6 YPT31(K127N)::URA3 GFP-VPS21::HIS3 msb3Δ::LEU2*	this study
JJTY4523	*Mat***a** *his3Δ1 leu2Δ0 ura3Δ0 lys2Δ0 GFP-VPS21(Q66L)::HIS3*	this study
JJTY4524	*Mat***a** *his3Δ1 leu2Δ0 ura3Δ0 lys2Δ0 GFP-VPS21(S21N)::HIS3*	this study
JJTY4580	*Mat***a** *his3Δ1 leu2Δ0 ura3Δ0 lys2Δ0 GFP-VPS21::HIS3 end3Δ::LEU2*	this study
JJTY4584	*Mat***a** *his3Δ1 leu2Δ0 ura3Δ0 met15Δ0 vps9Δ::KanMX6 GFP-VPS21::HIS3 muk1Δ::URA3*	this study
JJTY4696	*Mat***a** *his3Δ1 leu2Δ0 ura3Δ0 lys2Δ0 bar1Δ::HphMX4*	this study
JJTY5159	*Mat***a** *his3Δ1 leu2Δ0 ura3Δ0 trs120-3'UTR::KanMX6 GFP-VPS21::HIS3*	this study
JJTY5164	*Mat***a** *his3Δ1 leu2Δ0 ura3Δ0 trs120-3'UTR::KanMX6 GFP-VPS21::HIS3 msb3Δ::LEU2*	this study
JJTY5171	*Mat***a** *his3Δ1 leu2Δ0 ura3Δ0 lys2Δ0 GFP-VPS9::HIS3 (ZWF1 promoter)*	this study
JJTY5175	*Mat***a** *his3Δ1 leu2Δ0 ura3Δ0 lys2Δ0 GFP-VPS9::HIS3 (ZWF1 promoter) SEC7-mCherry::URA3*	this study
JJTY5176	*Mat***a** *his3Δ1 leu2Δ0 ura3Δ0 lys2Δ0 GFP-VPS9::HIS3 (ZWF1 promoter) HSE1-tdTomato::URA3*	this study

JJTY5198	Mata his3Δ1 leu2Δ0 ura3Δ0 trs120-3'UTR::KanMX6 GFP-YPT31::HIS3	this study
JJTY5199	Mata his3Δ1 leu2Δ0 ura3Δ0 trs120-3'UTR::KanMX6 GFP-YPT31(Q72L)::HIS3	this study
JJTY5227	Mata his3Δ1 leu2Δ0 ura3Δ0 lys2Δ0 GFP-VPS21::HIS3 VPH1-mCherry::URA3	this study
JJTY5511	Mata his3Δ1 leu2Δ0 ura3Δ0 lys2Δ0 GFP-VPS21::HIS3 HSE1-tdTomato::URA3	this study
JJTY6509	Mata his3Δ1 leu2Δ0 ura3Δ0 lys2Δ0 ypt32Δ::KanMX6 YPT31(K127N)::URA3 GFP-VPS9::HIS3 (ZWF1 promoter) SEC7-mCherry::HphMX4	this study
JJTY6510	Mata his3Δ1 leu2Δ0 ura3Δ0 lys2Δ0 ypt32Δ::KanMX6 YPT31(K127N)::URA3 GFP-VPS9::HIS3 (ZWF1 promoter) HSE1-tdTomato::HphMX4	this study
JJTY6519	Mata his3Δ1 leu2Δ0 ura3Δ0 met15Δ0 ent3Δ::KanMX6 GFP-VPS9::HIS3 (ZWF1 promoter) ent5Δ::LEU2	this study
JJTY6526	Mata his3Δ1 leu2Δ0 ura3Δ0 met15Δ0 ent3Δ::KanMX6 GFP-VPS9::HIS3 (ZWF1 promoter) ent5Δ::LEU2 SEC7-mCherry::URA3	this study
JJTY6527	Mata his3Δ1 leu2Δ0 ura3Δ0 met15Δ0 ent3Δ::KanMX6 GFP-VPS9::HIS3 (ZWF1 promoter) ent5Δ::LEU2 HSE1-tdTomato::URA3	this study
JJTY6559	Mata his3Δ1 leu2Δ0 ura3Δ0 lys2Δ0 erg6Δ::KanMX6 bar1Δ::LEU2	this study
JJTY6562	Mata his3Δ1 leu2Δ0 ura3Δ0 met15Δ0 vps9Δ::KanMX6 muk1Δ::URA3 bar1Δ::LEU2	this study
JJTY6763	Mata his3Δ1 leu2Δ0 ura3Δ0 lys2Δ0 GFP-VPS9::HIS3	this study
JJTY6765	Mata his3Δ1 leu2Δ0 ura3Δ0 met15Δ0 ent3Δ::KanMX6 GFP-VPS9::HIS3 ent5Δ::LEU2	this study
JJTY8566	Mata his3Δ1 leu2Δ0 ura3Δ0 met15Δ0 arf1Δ::KanMX6 GFP-VPS9::HIS3	this study
JJTY8567	Mata his3Δ1 leu2Δ0 ura3Δ0 lys2Δ0 GFP-VPS9::HIS3 (ZWF1 promoter) ARF1-BirA(R118G)-Stag::LEU2	this study
JJTY8568	Mata his3Δ1 leu2Δ0 ura3Δ0 lys2Δ0 ypt32Δ::KanMX6 YPT31(K127N)::URA3 SEC7-GFP::HIS3 ENT3-mCherry::HphMX4	this study
JJTY8569	Mata his3Δ1 leu2Δ0 ura3Δ0 lys2Δ0 ypt32Δ::KanMX6 YPT31(K127N)::URA3 SEC7-GFP::HIS3	

	ENT5-mCherry::*HphMX4*	this study
JJTY8570	*Mat***a** *his3Δ1 leu2Δ0 ura3Δ0 lys2Δ0 ypt32Δ*::*KanMX6 YPT31(K127N)*::*URA3 SEC7-GFP*::*HIS3 APL2-mCherry*::*HphMX4*	this study
JJTY8571	*Mat***a** *his3Δ1 leu2Δ0 ura3Δ0 lys2Δ0 bar1Δ*::*HphMX4 vps9Δ*CUE::*LEU2*	this study
JJTY8572	*Mat***a** *his3Δ1 leu2Δ0 ura3Δ0 lys2Δ0 arf1Δ*::*URA3 bar1Δ*::*HphMX4*	this study
JJTY8573	*Mat***a** *his3Δ1 leu2Δ0 ura3Δ0 lys2Δ0 arf1Δ*::*URA3 bar1Δ*::*HphMX4 vps9Δ*CUE::*LEU2*	this study
JJTY8574	*Mat***a** *his3Δ1 leu2Δ0 ura3Δ0 met15Δ0 arf1Δ*::*KanMX6 GFP-VPS9*::*HIS3 (ZWF1 promoter)*	this study
JJTY8575	*Mat***a** *his3Δ1 leu2Δ0 ura3Δ0 lys2Δ0 vps4Δ*::*KanMX6 GFP-VPS9*::*HIS3 (ZWF1 promoter) SEC7-mCherry*::*URA3*	this study
JJTY8576	*Mat***a** *his3Δ1 leu2Δ0 ura3Δ0 met15Δ0 arf1Δ*::*KanMX6 GFP-VPS9*::*HIS3 (ZWF1 promoter) vps4Δ*::*URA3*	this study
JJTY8638	*Mat***a** *his3Δ1 leu2Δ0 ura3Δ0 met15Δ0 arf1Δ*::*KanMX6 VPS8-GFP*::*HIS3*	this study
JJTY8639	*Mat***a** *his3Δ1 leu2Δ0 ura3Δ0 met15Δ0 ent3Δ*::*KanMX6 VPS8-GFP*::*HIS3 ent5Δ*::*LEU2*	this study
JJTY8650	*Mat***a** *his3Δ1 leu2Δ0 ura3Δ0 lys2Δ0 VPS8-GFP*::*HIS3 VPH1-mCherry*::*URA3*	this study
JJTY8657	*Mat***a** *his3Δ1 leu2Δ0 ura3Δ0 lys2Δ0 sac6Δ*::*KanMX6 VPS8-GFP*::*HIS3 scp1Δ*::*LEU2*	this study
JJTY9298	*Mat***a** *his3Δ1 leu2Δ0 ura3Δ0 met15Δ0 rom1Δ*::*KanMX6 VPH1-GFP*::*HIS3 scp1Δ*::*LEU2 sac6Δ*::*URA3*	this study
JJTY9299	*Mat***a** *his3Δ1 leu2Δ0 ura3Δ0 lys2Δ0 ARF1-GFP*::*HIS3 erg6Δ*::*URA3*	this study
RRS0050	*Mat***a** *his3Δ1 leu2Δ0 ura3Δ0 lys2Δ0 sac6Δ*::*KanMX6 GFP-VPS21*::*HIS3*	this study
RRS0051	*Mat***a** *his3Δ1 leu2Δ0 ura3Δ0 met15Δ0 arf1Δ*::*KanMX6 GFP-VPS21*::*HIS3*	this study
RRS0052	*Mat***a** *his3Δ1 leu2Δ0 ura3Δ0 met15Δ0 ent3Δ*::*KanMX6 GFP-VPS21*::*HIS3*	this study
RRS0053	*Mat***a** *his3Δ1 leu2Δ0 ura3Δ0 met15Δ0 ent3Δ*::*KanMX6 GFP-VPS21*::*HIS3 ent5Δ*::*LEU2*	this study
RRS0442	*Mat***a** *his3Δ1 leu2Δ0 ura3Δ0 lys2Δ0 sac6Δ*::*KanMX6 GFP-VPS21*::*HIS3 scp1Δ*::*LEU2*	this study
RRS0444	*Mat***a** *his3Δ1 leu2Δ0 ura3Δ0 met15Δ0 apl4Δ*::*KanMX6 GFP-VPS21*::*HIS3*	this study

RRS0445	*Mat***a** *his3Δ1 leu2Δ0 ura3Δ0 met15Δ0 gga1Δ*::*KanMX6 GFP-VPS21*::*HIS3 gga2Δ*::*LEU2*	this study
RRS0446	*Mat***a** *his3Δ1 leu2Δ0 ura3Δ0 met15Δ0 gga1Δ*::*KanMX6 GFP-VPS21*::*HIS3 gga2Δ*::*LEU2 apl4Δ*::*URA3*	this study
RRS0461	*Mat***a** *his3Δ1 leu2Δ0 ura3Δ0 lys2Δ0 erg6Δ*::*KanMX6 GFP-VPS21*::*HIS3*	this study
RRS0514	*Mat***a** *his3Δ1 leu2Δ0 ura3Δ0 met15Δ0 arf1Δ*::*KanMX6 GFP-VPS21(Q66L)*::*HIS3*	this study
RRS0540	*Mat***a** *his3Δ1 leu2Δ0 ura3Δ0 lys2Δ0 GFP-VPS21*::*HIS3 sla2Δ*::*LEU2*	this study
RRS0550	*Mat***a** *his3Δ1 leu2Δ0 ura3Δ0 lys2Δ0 myo3Δ*::*KanMX6 GFP-VPS21*::*HIS3 myo5Δ*::*LEU2*	this study
RRS0601	*Mat***a** *his3Δ1 leu2Δ0 ura3Δ0 met15Δ0 ent5Δ*::*KanMX6 GFP-VPS21*::*HIS3*	this study
RRS0602	*Mat***a** *his3Δ1 leu2Δ0 ura3Δ0 lys2Δ0 GFP-VPS21*::*HIS3 vps9Δ*CUE::*URA3*	this study
RRS0603	*Mat***a** *his3Δ1 leu2Δ0 ura3Δ0 met15Δ0 arf1Δ*::*KanMX6 GFP-VPS21*::*HIS3 vps9Δ*CUE::*URA3*	this study
RRS0604	*Mat***a** *his3Δ1 leu2Δ0 ura3Δ0 met15Δ0 arf1Δ*::*KanMX6 GFP-VPS21*::*HIS3 SEC7-mCherry*::*LEU2*	this study
RRS0731	*Mat***a** *his3Δ1 leu2Δ0 ura3Δ0 met15Δ0 ent3Δ*::*KanMX6 GFP-VPS21*::*HIS3 ent5Δ*::*LEU2 HSE1-tdTomato*::*HphMX4*	this study
RRS0773	*Mat***a** *his3Δ1 leu2Δ0 ura3Δ0 lys2Δ0 sac6Δ*::*KanMX6 GFP-VPS21*::*HIS3 scp1Δ*::*LEU2 HSE1-tdTomato*::*URA3*	this study
RRS0795	*Mat***a** *his3Δ1 leu2Δ0 ura3Δ0 met15Δ0 arf1Δ*::*KanMX6 GFP-VPS21*::*HIS3 HSE1-tdTomato*::*URA3*	this study
RRS0842	*Mat***a** *his3Δ1 leu2Δ0 ura3Δ0 met15Δ0 rom1Δ*::*KanMX6 GFP-VPS21*::*HIS3 scp1Δ*::*LEU2 sac6Δ*::*URA3*	this study
RRS0843	*Mat***a** *his3Δ1 leu2Δ0 ura3Δ0 lys2Δ0 GFP-VPS21*::*HIS3 bar1Δ*::*LEU2 erg6Δ*::*URA3*	this study
RRS0844	*Mat***a** *his3Δ1 leu2Δ0 ura3Δ0 lys2Δ0 GFP-VPS21*::*HIS3 erg6Δ*::*LEU2 SEC7-mCherry*::*URA3*	this study
RRS0845	*Mat***a** *his3Δ1 leu2Δ0 ura3Δ0 lys2Δ0 GFP-VPS21*::*HIS3 erg6Δ*::*LEU2 HSE1-tdTomato*::*URA3*	this study
